# Susceptibility of GT1–7 cells to mouse-passaged field scrapie isolates with a long incubation

**DOI:** 10.4161/pri.32232

**Published:** 2014-11-01

**Authors:** Kohtaro Miyazawa, Hiroyuki Okada, Yoshifumi Iwamaru, Kentaro Masujin, Takashi Yokoyama

**Affiliations:** Influenza and Prion Disease Research Center; National Institute of Animal Health; NARO; Tsukuba, Ibaraki, Japan

**Keywords:** cell susceptibility, prion, scrapie, strains

## Abstract

A typical feature of scrapie in sheep and goats is the accumulation of disease-associated prion protein. Scrapie consists of many strains with different biological properties. Nine natural sheep scrapie cases were transmitted to wild-type mice and mouse-passaged isolates were classified into 2 types based on incubation time: short and long. These 2 types displayed a distinct difference in their pathology. We attempted to transmit these mouse-passaged isolates to 2 murine cell lines (GT1–7 and L929) to compare their properties. All of the isolates were transmitted to L929 cells. However, only mouse-passaged field isolates with a long incubation time were transmitted to GT1–7 cells. This specific susceptibility of GT1–7 cells was also confirmed with a primary-passaged isolate that was not completely adapted to the new host species. Characterization of the mechanisms of the specific susceptibility of GT1–7 cells to isolates with a long incubation time may lead to a greater understanding of the differences among prion strains.

## Introduction

Scrapie is a transmissible spongiform encephalopathy (TSE) in sheep and goats. The central event of TSE pathogenesis is the accumulation of the disease-associated isoform of the prion protein (PrP^Sc^), which is thought to be the primary or sole component of the TSE agent (or prion).^1^ PrP^Sc^ is generated by post-translational modification of a host-encoded cellular prion protein (PrP^C^), but they have different conformational and physicochemical properties.^2,3^ The infectious agent itself remains enigmatic.^4^

Scrapie prions are classified into many biologically different strains and more than 20 strains have been reported.^5^ Prion strains are classified according to biological properties such as incubation time, histopathological lesion profile, and PrP^Sc^ deposition pattern in inbred mice.^6-8^ No scrapie prions have been transmitted to humans. However, the detection of bovine spongiform encephalopathy (BSE) in goats^9,10^ has increased the need for surveillance and strain-typing of natural scrapie.

Some prion strains can be discriminated by the biochemical properties of PrP^Sc^, such as molecular mass, glycoform ratio, proteinase K (PK) sensitivity,^11-13^ and conformation stability.^14,15^ However, some scrapie isolates have shown different biological properties irrespective of their similar PrP^Sc^ banding pattern.^16,17^

We examined the characteristics of natural sheep scrapie cases by mouse transmission. Ten mouse-passaged scrapie isolates were obtained and classified into 2 types. One had a short incubation period and caused prominent vacuolation in affected mice (short-type). In contrast, the other had a long incubation period and caused relatively mild vacuolation in affected mice (long-type).

Several cell lines are known to show susceptibility to mouse-adapted prions.^18-21^ By using these cell lines, a couple of methods have been developed for the discrimination of prion strains and the evaluation of prion infectivity.^22,23^ GT1–7 cells have been used in prion research and exhibit susceptibility to a variety of mouse-adapted prion strains and stable PrP^Sc^ replication.^24-27^ However, this cell line is insusceptible to the laboratory scrapie strain ME7.^28^ In contrast, L929 cells exhibit susceptibility to several laboratory scrapie strains, including ME7.^28^

In this study, we examined the susceptibility of GT1–7 cells and L929 cells to 2 types of mouse-passaged scrapie isolates. Both types of isolates were transmitted to L929 cells. In contrast, long-type isolates were transmitted to GT1–7 cells, but short-type isolates were not. This difference in transmissibility to GT1–7 cells was confirmed not only for prions from the subsequent passaged mice (2nd, 3rd or serial passage), but also in primary passaged mice.

## Results

### Classification of natural sheep scrapie isolates into 2 types

**Figure 1.**
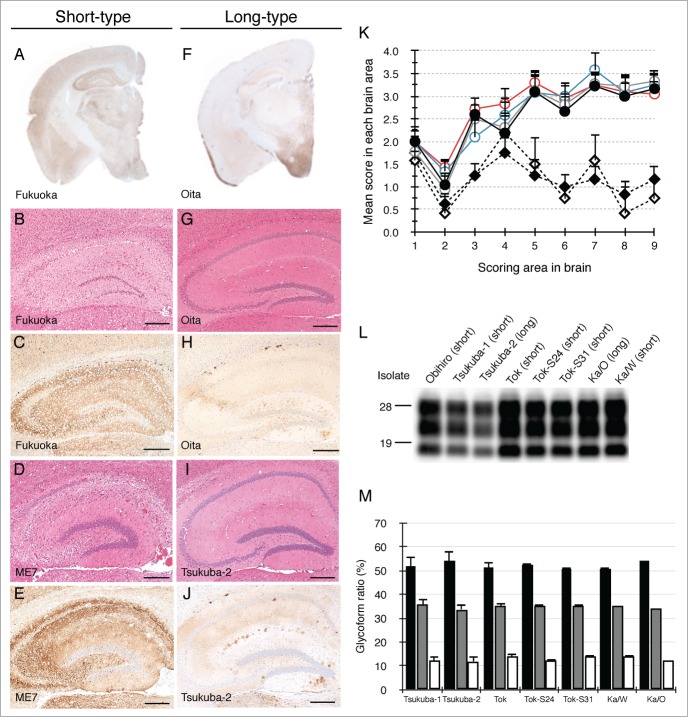
Characterization of 2 types of mouse-passaged scrapie isolates. Immunohistochemical detection of PrP^Sc^ was performed using the monoclonal antibody (mAb) SAF84. The coronal sections at the level of the hippocampus of brains affected with a short-type isolate (**A**) and a long-type isolate (**F**) are shown, respectively. Sections of the hippocampus of mice affected with Fukuoka (**B and C**), ME7 (**D and E**), Oita (**G and H**) and Tsukuba-2 (**I and J**) were subjected to H&E staining (**B, D, G and I**) and immunostaining of PrP^Sc^ (**C, E, H and J**). The bars represent 200 μm. Lesion profiles of mice affected with ME7, Obihiro, Tok, Fukuoka, Tsukuba-2 and Oita in 9 brain areas are shown (**K**). The brain vacuolation of mice affected with ME7 (closed circle, n = 6), Obihiro (gray opened circle, n = 6), Tok (blue opened circle, n = 3), Fukuoka (red opened circle, n = 6), Tsukuba-2 (closed diamond, n = 5), and Oita (opened diamond, n = 6) were scored on a scale of 0 to 5 (mean value + standard deviation). Serially passaged brains of ME7- and Obihiro-affected mice, second-passaged brains of Tok-, Fukuoka- and Oita-affected mice, and third-passaged brains of Tsukuba-2-affected mice were subjected to the scoring. The different brain areas are indicated as follows: 1 dorsal medulla, 2 cerebellar cortex, 3 superior cortex, 4 hypothalamus, 5 thalamus, 6 hippocampus, 7 septal nuclei of the paraterminal body, 8 cerebral cortex at the levels of the hypothalamus and the thalamus, 9 cerebral cortex at the level of the septal nuclei of the paraterminal body. Representative western blot of PrP^Sc^ in mice affected with a variety of mouse-passaged field scrapie isolates is shown (**L**). The name of the isolates and respective grouping into short- and long-type are indicated on the top of each lane. Total protein (10 μg) was loaded per lane and probed with mAb SAF84 to detect PrP^Sc^. Molecular markers are indicated on the left side of the panel. Glycoform profiles of PrP^Sc^ in mouse brains affected with 7 mouse-passaged scrapie isolates (M). PrP^Sc^ was detected with mAb SAF84. Signal intensities of di-, mono-, and non-glycosylated PrP^Sc^ bands were analyzed. Black bar: ratio of diglycosylated PrP^Sc^ to total PrP^Sc^. Gray bar: ratio of mono-glycosylated PrP^Sc^ to total PrP^Sc^. White bar: ratio of non-glycosylated PrP^Sc^ to total PrP^Sc^. Values are expressed as the mean ± standard deviation (%).

Nine Japanese natural scrapie cases were transmitted to wild-type (ICR) mice and 10 isolates were obtained. These isolates were categorized into 2 types based on their biological properties. One type displayed a short incubation time, marked vacuolation and widespread PrP^Sc^ distribution throughout the brain in mice (designated as short-type). Seven isolates (Obihiro,^29^ Tsukuba-1,^16^ Tok,^30^ Tok-S24, Tok-S31, Ka/W^17^ and Fukuoka) were classified as short-type isolates (**Table 1**). Based on their incubation times, the lesion profiles and the distribution of PrP^Sc^, these isolates had characteristics reminiscent of ME7 (**Fig. 1B–E**, **K** and **Table 1**). The other type displayed polydipsia and polyuria with a long incubation time in diseased mice (designated as long-type). Three isolates, (Tsukuba-2,^16^ Ka/O^17^ and Oita) were classified as long-type isolates (**Table 1**). The lesion profiles of mice affected with long-type isolates were obviously different from those of mice affected with short-type isolates (**Fig. 1G, I and K**). Mice affected with short-type isolates displayed fine and coarse granular PrP^Sc^ and prominent vacuolation throughout the brain (**Fig. 1A–E**). In contrast, PrP^Sc^ of mice affected with long-type isolates were mainly observed in the thalamus, hypothalamus, and brainstem and some PrP^Sc^ plaques were seen in the corpus callosum (**Fig. 1F–J**). In spite of different biological properties, there was no difference in PrP^Sc^ banding pattern between short-type and long-type isolates (**Fig. 1L and M**).

**Table 1.** Mouse-adapted field scrapie isolates used in this study

Case	Breed (PrP genotype)^b^	Isolate or strain (Phenotype)	Passage history in ICR mice	Incubation time	Transmissibility to GT1–7	Transmissibility to L929
Natural	Suffolk (NT ^c^)	Obihiro (Short-type)	Serial	141 ± 11.7 (8/8 ^d^)	–	+^e^
Natural	Corriedale (NT)	Tsukuba-1 (Short-type)	1st 2nd 3rd	213 ± 16 (6/6) 180 ± 8.0 (6/6) 133 ± 2.0 (6/6)	NT NT –	NT NT +^e^
Natural	Suffolk (NT)	Tsukuba-2 (Long-type)	1st 2nd 3rd	359 ± 21 (6/6) 299 ± 1.0 (6/6) 288 ± 5.0 (6/6)	NT NT +	NT NT NT
Natural	Suffolk (ARQ)	Tok (Short-type)	1st 2nd 3rd	295 ± 40 (11/11) 146 ± 1.0 (7/7) 146 ± 5.0 (10/10)	NT NT –	NT NT + ^e^
Natural	Suffolk (ARQ)	Tok-S24 (Short-type)	1st 2nd 3rd	426 ± 35 (6/6) 138 ± 1.0 (7/7) 146 ± 13 (7/7)	NT NT –	NT NT NT
Natural	Suffolk (ARQ)	Tok-S34 (Short-type)	1st 2nd 3rd	268 ± 24 (9/9) 155 ± 8.0 (5/5) 147 ± 0.5 (7/7)	NT NT –	NT NT NT
Natural ^a^	Suffolk (ARQ)	Ka/W (Short-type)	1st 2nd 3rd	457 ± 21 (15/16) 256 ± 29 (5/5) 152 ± 5.6 (10/10)	NT NT –	NT NT +
		Ka/O (Long-type)	1st 2nd 3rd	469 (1/16) 287 ± 6.5 (5/5) 272 ± 29.0 (10/10)	NT NT +	NT NT +
Natural	Suffolk (ARQ)	Fukuoka (Short-type)	1st 2nd	442 ± 62.4 (20/20) 155 ± 4.4 (10/10)	– –	NT +
Natural	Suffolk (ARQ)	Oita (Long-type)	1st 2nd	447 ± 33.7 (20/20) 279 ± 19.7 (10/10)	+ +	NT +
Laboratory strain		ME7	Serial	143 ± 4.4	−	+
Laboratory strain		Chandler	Serial	135 ± 5.6	+	NT
Laboratory strain		22L	Serial	138 ± 5.0	+	+^e^

a Two biologically different prions were isolated from one sheep. Sixteen mice were inoculated with scrapie-affected sheep brain homogenate. Only one mouse in the line of Ka/O first passage exhibited polydipsia, polyuria, and obesity as clinical signs while the others (15/16) did not show these clinical signs.

b PrP amino acid sequence at codons 136, 154, and 171.

c Not tested.

d Number of sick mice/total inoculated mice.

e Data not shown.

### Transmission study of mouse-passaged field scrapie isolates to GT1–7 and L929 cell lines

**Figure 2.**
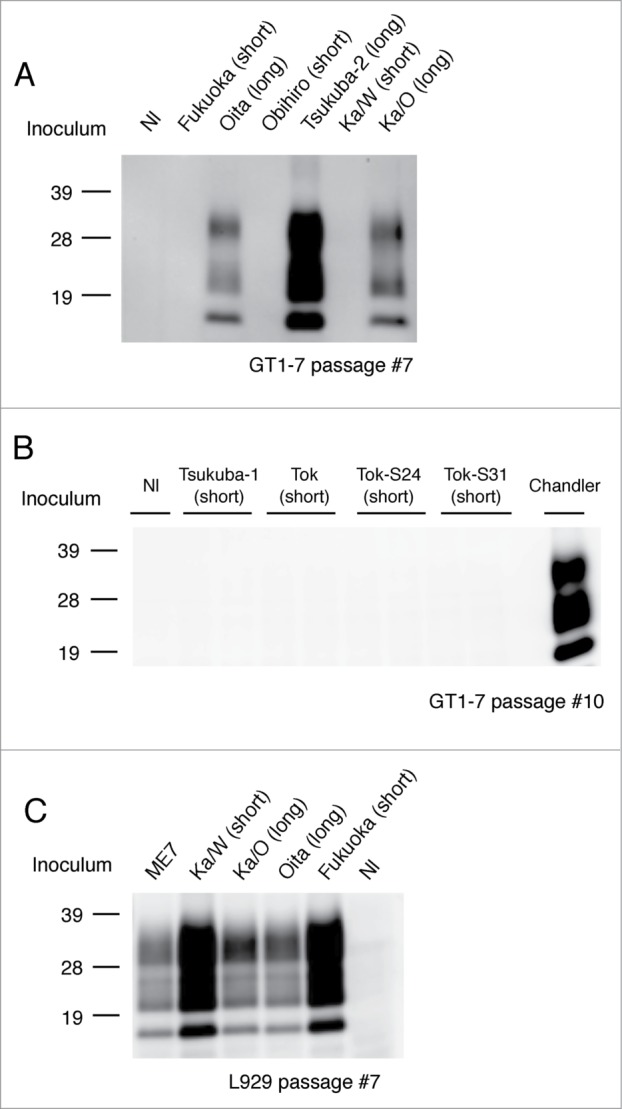
Transmission of short-type and long-type isolates to murine cell lines. Representative protein gel blots of GT1–7 cells exposed to short-type and long-type isolates are shown (**A and B**). The name of the isolate used for the challenge and respective grouping into short- and long-type are indicated on the top of each lane. Nl indicates the use of an uninfected mouse brain homogenate as the inoculum. Chandler strain was used for the positive control of the GT1–7 infection. Representative western blot of L929 cells exposed to short-type and long-type isolates is shown (**C**). The name of the isolate used for the challenge and respective grouping into short- and long-type are indicated on the top of each lane. Total protein (500 μg) was loaded into each lane except for the lane of Chandler (80 μg). The presence of PrP^Sc^ was analyzed after 7 passages and more to eliminate the influence of PrP^Sc^ derived from the brain homogenates. PrP^Sc^ was detected with mAb T2. Molecular markers are indicated on the left side of each panel.

In order to ascertain the transmissibility of the 2 types of isolates to murine cell lines, diseased mouse brain homogenates were exposed to GT1–7 cells and L929 cells, respectively. Interestingly, all the examined long-type isolates (Tsukuba-2,^16^ Ka/O^17^ and Oita) were successfully transmitted to GT1–7 cells and synthesized *de novo* PrP^Sc^ (**Fig. 2A**), whereas all the examined short-type isolates were not transmitted, except for the laboratory strains Chandler and 22L (**Fig. 2A–B** and **Table 1**). In contrast, L929 cells were susceptible to both short-type (Ka/W and Fukuoka) and long-type (Ka/O and Oita) isolates and synthesized *de novo* PrP^Sc^ (**Fig. 2C** and **Table 1**).

### Specific detection of long-type isolates in primary-passaged mice by GT1–7 cells

**Figure 3.**
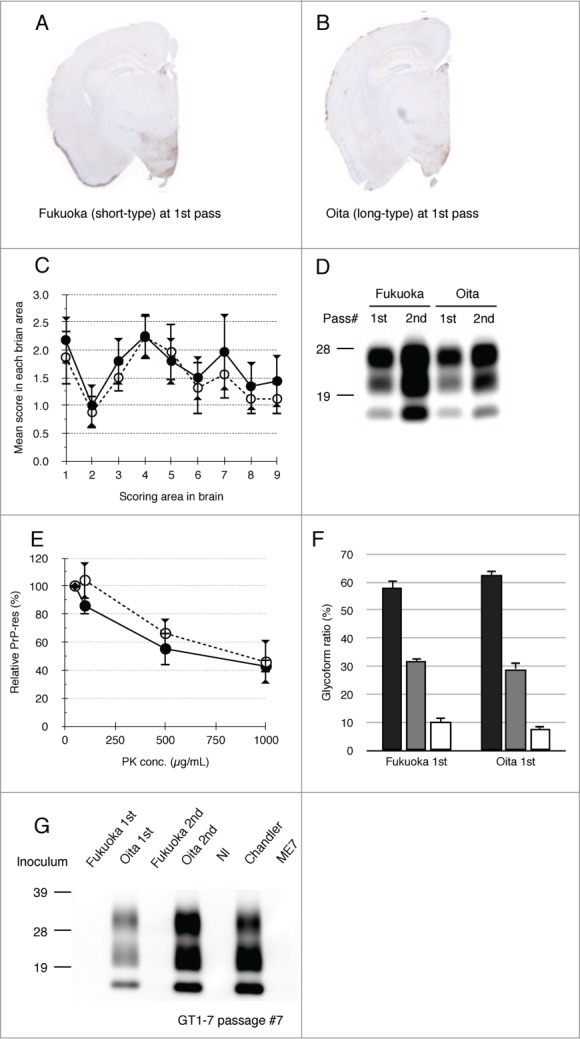
Biochemical and biological properties of short- and long-type prions in primary-passaged mouse brains. PrP^Sc^ distributions of primary-passaged Fukuoka and Oita affected mice are shown, respectively (**A and B**). Immunostaining was performed using the mAb SAF84. The coronal sections at the level of the hippocampus are shown. Lesion profiles of Fukuoka- and Oita-affected mice in 9 brain areas at first passage (**C**). The brain vacuolation of Fukuoka- (closed circle) and Oita-affected mice (opened circle) was scored on a scale of 0 to 5 (mean value ± standard deviation). The brain areas are as indicated in **Figure 1**. Representative protein gel blot of PrP^Sc^ in Fukuoka- and Oita-affected mice (**D**). The name of the isolate and passage number in mice are indicated on the top of panel. For each sample, 10 μg of total protein was loaded per lane and probed with mAb SAF84 to detect PrP^Sc^. Molecular markers are indicated on the left side. PK sensitivity of PrP^Sc^ between Fukuoka- and Oita-affected mice at first passage is compared (**E**). Closed circles and opened circles represent the results of Fukuoka- and Oita-affected mice, respectively. PrP^Sc^ was detected with mAb 6H4. Data represent the mean ± standard deviation from 4 to 5 independent experiments. Glycoform profiles of PrP^Sc^ in Fukuoka- and Oita-affected mice at first passage (**F**). PrP^Sc^ was detected with mAb 6H4. Signal intensities of di-, mono-, and non-glycosylated PrP^Sc^ bands were analyzed. Black bar: ratio of diglycosylated PrP^Sc^ to total PrP^Sc^. Gray bar: ratio of mono-glycosylated PrP^Sc^ to total PrP^Sc^. White bar: ratio of non-glycosylated PrP^Sc^ to total PrP^Sc^. Values are expressed as the mean ± standard deviation (%). Representative western blot of GT1–7 cells exposed to Fukuoka (short-type isolate) and Oita (long-type isolate) is shown (**G**). Inocula used in this study are indicated on the top of each lane. Nl indicates the use of an uninfected mouse brain homogenate as the inoculum. PrP^Sc^ was detected by mAb T2. Molecular markers are indicated on the left side of panel. Total protein (500 μg) was loaded into each lane except for the lane of Chandler (80 μg).

The specific response of GT1–7 cells against long-type isolates was examined using primary-passaged mouse brains affected with Fukuoka or Oita scrapie. Both scrapie isolates were transmissible to ICR mice with a 100% attack rate and their incubation times were 442 ± 62.4 d (Fukuoka) and 447 ± 33.7 d (Oita), respectively, at primary passage (**Table 1**). As shown in **Figure 3A and B**, the distribution patterns of PrP^Sc^ in the brains of the mice affected with these 2 isolates were alike and PrP^Sc^ was mainly detected in the hypothalamus of both isolates of scrapie-affected mice. Neuropathological analysis also demonstrated similar lesion profiles in primary-passaged mice (**Fig. 3C**). In addition, both isolates displayed indistinguishable biochemical properties of PrP^Sc^, including PK-sensitivity, glycoform profile, and conformational stability (**Fig. 3D–F** and **Table 2**). No clear difference was observed in the primary-passaged mice of Fukuoka and Oita scrapie. However, subsequent passages revealed differences in their biological properties: Fukuoka isolate was classified as a short-type and Oita isolate was classified as a long-type isolate (**Fig. 1A–C, F–H** and **Table 1**).

**Table 2.** Conformation stability of PrP^Sc^ of mice affected with Fukuoka and Oita at primary passage

Isolate (Phenotype)	[GdnHcl]_1/2_
Fukuoka (Short-type)	2.17 ± 0.22
Oita (Long-type)	2.16 ± 0.16

[GdnHCl]_1/2_ (M) was calculated based on the sigmoidal dose-response curve. The results are shown as mean ± standard deviation. No significant difference in [GdnHCl]_1/2_ was observed between the 2 strains by the Student's *t* test (*P* < 0.05).

As shown in **Figure 3G**, PrP^Sc^ was detected from Oita-challenged GT1–7 cells at primary and secondary passage (lane 2 and 4). In contrast, no PrP^Sc^ was detected from Fukuoka-challenged cells (lane 1 and 3). GT1–7 cells were preferably permissive only to the Oita isolate and produced *de novo* PrP^Sc^, irrespective of their passage numbers. These results indicate that the transmissibility of mouse-passaged isolates to cell lines is determined prior to their biological adaptation to the new host.

## Discussion

Prion strain typing is based on the incubation time and lesion profile of mouse-adapted isolates.^5,7^ In the interspecies transmission of prions, a couple of passage histories are required to stabilize properties such as incubation time, brain pathology, and topology of accumulated PrP^Sc^.^5,12^ In this study, we transmitted 9 natural sheep scrapie cases to ICR mice to explore the extent of phenotypic variation and obtained 10 mouse-passaged isolates. No distinct difference was observed in PrP^Sc^ banding pattern and glycoprofile among all isolates. Thus, to distinguish these isolates, biological analysis using mouse bioassays were necessary. The isolates were categorized into 2 types. The short-type isolates that were examined were all reminiscent of ME7 by their biological characterization (**Fig. 1**). The incubation times of the laboratory scrapie strains 22L and Chandler in ICR mice were approximately 140 d (**Table 1**) and were similar to those of short-type isolates in this study. GT1–7 cells are known to be insusceptible to ME7, but susceptible to 22L and Chandler.^28^ Therefore, the insusceptibility of GT1–7 cells to the examined short-type isolates also suggests similarity of these Japanese isolates with ME7.

Although present and previous studies used different mouse lines with the same PrP allele (ICR and C57BL), the pathology of mice affected with long-type isolates was similar with the reported pathology of mouse-passaged scrapie strain 87A.^31,32^ There is no report regarding the susceptibility of GT1–7cells to mouse-passaged scrapie isolates with a long incubation time. Our results indicate the possibility that GT1–7 cells may be susceptible to 87A. Indeed, it has been reported that GT1–7 cells are susceptible to a mouse-passaged sporadic Creutzfeldt-Jakob isolate that shows a long incubation time in ICR mice.^33^ This cell line will be a useful tool for the analysis of slowly propagated prions in the *in vivo* environment.

GT1–7 cells could amplify PrP^Sc^ of a long-type isolate from the primary-passaged mice, even though the isolate did not yet adapt to the new host. Furthermore, mice affected with long-type isolates showed pathology similar to mice affected with short-type isolates at this stage (**Fig. 3**). A PrP^Sc^ tracing experiment using a conformation-specific monoclonal antibody revealed that the PrP^Sc^ structure was altered during prion adaptation to the new host animals.^34^ Our study demonstrated that the susceptibility of prions to GT1–7 cells was independent from this conformational transition of PrP^Sc^ in interspecies transmission. This isolate-specific response of GT1–7 cells will be useful as a time- and cost-saving alternative to the mouse bioassay.

Since ICR mice, GT1–7 cells and L929 cells express the same PrP alleles, the difference in susceptibility to long-type isolates between GT1–7 cells and L929 cells is not associated with the amino acid sequence of PrP. Though the precise mechanisms of the altered susceptibility of GT1–7 cells to some mouse-passaged scrapie isolates are unknown, conformational differences of PrP^Sc^ among strains and/or isolates are considerable. The different susceptibility of GT1–7 cells to RML and ME7 could be accounted for by the different conformation of PrP^Sc^.^14^ Another technique will be necessary to detect the structural differences of PrP^Sc^ between long-type and short-type isolates. Another possibility is structural differences of PrP^C^ between GT1–7 cells and L929 cells. It also may link to the PrP^Sc^ tropisms in mouse prion strains. The different origin of 2 cell lines, hypothalamic neurons (GT1–7 cells)^35^ and fibroblasts (L929 cells)^20^ may contribute to this difference.

*In vitro* classification of prion strains by the use of several cell lines has been reported.^36,37^ Our results also indicate that the isolate-specific response of GT1–7 cells could work from the primary-passaged mice. The specific amplification of prion in the cell culture may be useful for the cloning and/or isolation of a strain. Clarification of the molecular mechanisms involved in this phenomenon may lead to a better understanding of prion pathogenesis.

## Materials & Methods

### Scrapie sources

The mouse-passaged field scrapie isolates used in this study are listed in **Table 1**. The details of some of those isolates were described in previous reports.^16,17,29,30^ Rodent-passaged laboratory scrapie strains (Chandler, 22L and ME7) were maintained by serial-passage into wild-type mice (ICR; Japan SLC, Hamamatsu, Japan).^38^ All experiments involving animals were approved by the Animal Ethical Committee and the Animal Care and Use Committee of the National Institute of Animal Health (authorization 11–008 and 11–012).

### Scrapie transmission to wild-type mice

Twenty microliter of 10% (w/v) brain homogenate from the diseased sheep was intracerebrally inoculated into 3-week-old female ICR mice. Mouse brains were collected at the terminal stage of the disease or after death and subjected to biological and biochemical analyses and subsequent transmission to wild-type mice. Incubation was measured as the time between inoculation and the clinical endpoint or death. All brains of euthanized and dead mice were examined for the presence of PrP^Sc^ by Western blot.

### Transmission of mouse-passaged field scrapie isolates to GT1–7 and L929 cell lines

GT1–7 cells were maintained and exposed to mouse- passaged scrapie prions as described previously.^39^ Briefly, cells were grown on 6-well plates at 10^5^ cells/well. After 2 d of incubation, cells were exposed to 0.1% (w/v) brain homogenate in medium (1 mL) for 1 day. Additionally, 1 mL of plain medium was added to each well. Cells were incubated for 1 day and seeded into new 6-well plates for the first passage (P1). L929 cells were maintained in Dulbecco's modified eagle medium adding 10% fetal bovine serum and exposed to mouse- passaged scrapie prions as described above. For subsequent passages, GT1–7 and L929 cells were seeded at a 1:4 and 1:10 ratio every 4 days, respectively. In order to eliminate the influence of PrP^Sc^ in the exposed brain homogenate and stabilize PrP^Sc^ accumulation in cells, the presence of PrP^Sc^ in cells was analyzed after 7 passages and more.

### Histopathology and immunohistochemistry

The right lobes of brains were fixed in 10% neutral buffered formalin (pH 7.4) containing 10% methanol overnight at 37°C. Coronal slices of the brains were immersed in 98% formic acid for 60 min at room temperature (RT) to reduce infectivity and then embedded in paraffin wax. Serial sections were stained with hematoxylin and eosin (H&E) for neuropathological changes including the lesion profile^6^ or for immunohistochemistry. After epitope retrieval, PrP^Sc^ immunohistochemistry was performed using the mouse monoclonal antibody (mAb) SAF84 (Bertin Pharma, Montigny le Bretonneux, France).^40^ Immunoreactions were developed using the anti-mouse, universal immunoperoxidase polymer (Nichirei Histofine Simple Stain MAX-PO (M); Nichirei, Tokyo, Japan) as the secondary antibody and were visualized with 3,3′-diaminobenzedine tetrachloride as the chromogen.

### PrP^Sc^ detection from brains and cells

Brains were homogenized in 4 volumes of buffer containing 150 mM NaCl and 50 mM Tris-HCl (pH 7.6) to obtain 20% w/v, and were stored at −80°C until use. For the detection of PrP^Sc^, 250 μL of 5% (w/v) brain homogenate was mixed with an equal volume of detergent buffer containing 4% (w/v) Zwittergent 3–14, 1% (w/v) Sarkosyl, 100 mM NaCl, and 50 mM Tris-HCl (pH 7.6), and then treated with 1.6 μL of 80 mg/mL collagenase and 1 μL of 10 mg/mL DNase I for 30 min at 37°C. To digest PrP^C^, the sample was treated with 1 μL of 20 mg/mL PK (40 μg/mL final concentration) (Roche Diagnostics, Basel, Switzerland) for 30 min at 37°C then the digestion was terminated by adding 2.5 μL of 0.4 M 4-(2-aminoethyl)-benzenesulfonyl fluoride hydrochloride (Pefablock; Roche Diagnostics). The sample was then mixed with a 2-butanol: methanol mixture (5:1) and centrifuged at 20,000 *× g* for 10 min at RT. The pellet was resuspended in SDS-sample buffer and boiled for 5 min. Then, the samples were then subjected to western blot analysis.

In order to detect PrP^Sc^ in cells, confluent cells were washed with PBS and then lysed with lysis buffer containing 50 mM Tris-HCl [pH 7.6], 150 mM NaCl, 0.5% (w/v) Triton X-100, 0.5% (w/v) sodium deoxycholate, and 5 mM EDTA. After 2 min of centrifugation at 6,500 *× g* at 4°C, the supernatant was collected and the total protein concentration was measured using the Bio-Rad protein assay (Bio-Rad Laboratories, Hercules, CA). The protein concentration of each cell lysate was adjusted to 1 mg/mL and the samples were treated with 2 μL of 20 mg/mL PK (40 μg/mL final concentration) at 37°C for 30 min. Following PK digestion, the samples were mixed with a 2-butanol: methanol mixture (5:1) and centrifuged at 20,000 *× g* for 10 min at RT. The pellet was solubilized in SDS-sample buffer and boiled for 5 min. The samples were then subjected to Western Blot analysis.

Brain and cell samples were loaded onto a 12% polyacrylamide gel. The proteins were transferred onto an Immobilon-P membrane (Millipore, Billerica, MA). The blotted membrane was incubated with anti-PrP mAbs T2,^41^ 6H4 (Prionics, Zurich, Switzerland) and SAF84 at 4°C overnight. Following twice washing with PBST, the membrane was incubated with horseradish peroxidase (HRP)-conjugated anti-mouse IgG (Jackson ImmunoResearch, West Grove, PA) for 1 h at RT. Signals were developed with a chemiluminescent substrate (SuperSignal; Thermo Fisher Scientific, Rockland, IL). Blots were imaged with Fluochem (Alpha Innotech, San Leandro, CA) and analyzed with ImageReader software (AlphaEaseFC; Alpha Innotech) according to the manufacturer's instructions.

### PK-sensitivity assay

Brain samples were incubated with various PK concentrations (50, 100, 500, and 1000 μg/mL) at 37°C for 30 min. Samples were subjected to Western Blot as described above. PrP^Sc^ signals were detected with mAb 6H4 and normalized by the signal intensity of a 50 μg/mL PK-treated sample.

## 

### Conformational Stability Assay

The conformational stability assay was performed as previously described.^42^ Briefly, 25 μL of 10% (w/v) brain homogenate was added to an equal volume of guanidine hydrochloride (GdnHCl) in the concentration range of 1–8 M. Samples were mixed well and incubated at 37°C for 1 h. Then, the sample was diluted by the addition of 425 μL of a buffer containing 10 mM Tris-HCl (pH 8.0), 150 mM NaCl, 0.5% (w/v) Triton-X 100, and 0.5% (w/v) deoxycholate. Then, 25 μL of GdnHCl was added to each sample to a final concentration of 0.4 M. Following GdnHCl treatment, samples were digested with 20 μg/mL PK at 37°C for 1 h. PrP^Sc^ was precipitated and subjected to Western Blot analysis. PrP^Sc^ signals were detected using mAb SAF84 and normalized by the signal intensity of the 0.5 M GdnHCl^-^treated sample. The data were fitted into a sigmoidal dose-response curve using the KaleidaGraph software (Synergy Software, Reading, PA). Then, the half maximal effective concentrations of GdnHCl ([GdnHCl]_1/2_, M) were calculated.
